# Adhesive Property of 3D-Printed PEEK Abutments: Effects of Surface Treatment and Temporary Crown Material on Shear Bond Strength

**DOI:** 10.3390/jfb13040288

**Published:** 2022-12-09

**Authors:** Dingjie Wang, Xingting Han, Feng Luo, Florian M. Thieringer, Yichen Xu, Guomin Ou, Sebastian Spintzyk

**Affiliations:** 1 State Key Laboratory of Oral Diseases & National Clinical Research Center for Oral Diseases, Department of Oral Implantology, West China Hospital of Stomatology, Sichuan University, Chengdu 610041, China; 2 Department of Prosthodontics, Peking University School and Hospital of Stomatology, National Center of Stomatology, National Clinical Research Center for Oral Diseases, National Engineering Laboratory for Digital and Material Technology of Stomatology, Beijing Key Laboratory of Digital Stomatology, NHC Key Laboratory of Digital Technology of Stomatology, 22 Zhongguancun Avenue South, Beijing 100081, China; 3 State Key Laboratory of Oral Diseases & National Clinical Research Center for Oral Diseases, Department of Oral Prosthodontics, West China Hospital of Stomatology, Sichuan University, Chengdu 610041, China; 4 Medical Additive Manufacturing Research Group, Hightech Research Center, Department of Biomedical Engineering, University of Basel, 4123 Allschwil, Switzerland; 5 Department of Oral and Cranio-Maxillofacial Surgery, University Hospital Basel, 4031 Basel, Switzerland; 6 ADMiRE Research Center—Additive Manufacturing, Intelligent Robotics, Sensors and Engineering, School of Engineering and IT, Carinthia University of Applied Sciences, 9524 Villach, Austria

**Keywords:** fused filament fabrication, polyether ether ketone, additive manufacturing, shear bond strength, temporary abutment, dental implantology, rapid manufacturing, implant supported restoration

## Abstract

Three-dimensionally printed polyetheretherketone (PEEK) materials are promising for fabricating customized dental abutments. This study aimed to investigate the adhesive property of a 3D-printed PEEK material. The effects of surface treatment and temporary crown materials on shear bond strength were evaluated. A total of 108 PEEK discs were 3D printed by fused-filament fabrication. Surface treatments, including sandblasting, abrasive paper grinding, and CO_2_ laser ablation, were applied to the PEEK discs, with the untreated specimens set as the control. Afterward, the surface topographies of each group were investigated by scanning electron microscopy (SEM, *n* = 1) and roughness measurements (*n* = 7). After preparing the bonding specimens with three temporary crown materials (Artificial teeth resin (ATR), 3M™ Filtek™ Supreme Flowable Restorative (FR), and Cool Temp NATURAL (CTN)), the shear bond strength was measured (*n* = 6), and the failure modes were analyzed by microscopy and SEM. The results showed that ATR exhibited a significantly higher shear bond strength compared to FR and CTN (*p* < 0.01), and the PEEK surfaces treated by sandblasting and abrasive paper grinding showed a statistically higher shear bond strength compared to the control (*p* < 0.05). For clinical application, the ATR material and subtractive surface treatments are recommended for 3D-printed PEEK abutments.

## 1. Introduction

Implant abutments are sophisticated parts used for connecting the prosthesis and the implant. Nowadays, immediate and early restoration after implant surgery using temporary abutment and prosthesis is encouraged for its positive effect in guiding soft tissue healing and maintaining aesthetic function [1,2,3]. Although premade temporary abutments might be provided in different dimensions by manufacturers [4], these abutments can hardly fulfill the various prosthodontic requirements in a tooth defect area. Dentists and dental technicians have to spend extra time adjusting the temporary abutments, which significantly decreases medical efficiency [5,6].

With the rapid development of digital technologies in dentistry, computer-aided design (CAD) and additive manufacturing (AM, also known as 3D printing) may provide dentistry with customized solutions [7,8]. According to the operation plan, custom temporary abutments could be designed and fabricated in advance and placed right after surgery [9,10]. Fused filament fabrication (FFF) is one of the most popular 3D printing technologies that show prospects in abutment fabrication due to its advantages in rapid manufacturing and low costs [11]. By melting and extruding, FFF printers can deposit thermoplastic materials onto a build platform, constructing objects layer by layer [12].

Although there might be plenty of material choices for FFF, such as polylactic acid (PLA), acrylonitrile butadiene styrene (ABS), polyethylene terephthalate glycol (PETG), etc., polyetheretherketone (PEEK) seems to be a better choice for biomedical use due to its outstanding chemical resistance, excellent mechanical strength, and superb biocompatibility [13,14,15]. However, for temporary abutment applications, another aspect that must not be neglected is the material’s adhesive property because even a small detachment between the temporary abutment and crown may lead to prosthetic failure, which is not acceptable for both patients and dentists.

Previous studies have indicated that it is difficult to achieve firm adhesion with PEEK due to its hydrophobic nature and low surface energy [16]. Accordingly, various protocols have been investigated to enhance the adhesive ability of PEEK. Surface modifications, including sandblasting, sulfuric acid etching, and atmospheric plasma treatment, were reported to be effective [17,18,19]. However, less is known about the adhesive property of the 3D-printed PEEK material.

Therefore, the present study aimed to investigate the adhesive property of 3D-printed PEEK used for temporary abutment fabrication. The effects of surface treatment (sandblasting, abrasive paper grinding, and CO_2_ laser ablation) and the temporary crown material (Artificial teeth resin, 3M™ Filtek™ Supreme Flowable Restorative, and Cool Temp NATURAL) on the shear bond strength were evaluated. The null hypotheses were set as (i) the surface treatments do not affect the shear bond strength, and (ii) the temporary crown materials have no effect on the shear bond strength.

## 2. Materials and Methods

### 2.1. Specimen Preparation

The test specimen was designed as a disk with a diameter of 14 mm and a thickness of 2 mm using open-source CAD software (OpenSCAD, 2021.01, http://www.openscad.org/, accessed on 1 March 2022). A total of 108 specimens were 3D printed horizontally with a layer thickness of 200 µm by a FFF printer (Apium P220, Apium Additive Technologies GmbH, Karlsruhe, Germany) using a PEEK filament (Evonik VESTAKEEP^®^i4 G resin, Evonik Industries AG, Essen, Germany) [20]. After printing, the support structures were removed, and specimens were collected in zipper storage bags before testing.

### 2.2. Surface Treatment

Three surface treatments (sandblasting, abrasive paper grinding, and CO_2_ laser ablation) were included in this study (*n* = 27). The untreated PEEK specimens were set as the control. For the sandblasting group, the specimens were blasted with 100 μm aluminum oxide abrasive (Al_2_O_3_, DenTal DENTURE, Chengdu, China) by a sandblasting device (R-603, Zaofeng Technology Co., Ltd., Zhongshan, China) at a distance of 10 mm with a pressure of 0.2 MPa for 10 s. For the abrasive paper group, the specimens were ground with P600 silicon carbide abrasive paper (Suisun Company Limited, Hong Kong, China) under running cooling water for 60 rounds. Regarding the CO_2_ laser group, the PEEK specimens were ablated by a CO_2_ laser device (JZ-2, Beijing Health Medical Technology Co., Ltd., Beijing, China). The wavelength, frequency, rated input power, and point output power were set as 10.6 ± 0.1 μm, 50 Hz, 450 W, and 30 W, respectively. The diameter of each dot spaced 500 µm apart in the CO_2_ laser array was 100 µm. After surface treatment, all specimens were cleaned with compressed air for 20 s to remove material residues.

### 2.3. Surface Characterization

After surface treatment, the PEEK samples (*n* = 2) of each group were pictured under a stereoscopic microscope (SZX16, Olympus, Tokyo, Japan) at 1× and 4× magnifications and observed by a scanning electron microscope (SEM, JSM-IT500, JEOL, Tokyo, Japan) at 100×, 1000×, and 5000× magnifications, with an acceleration voltage of 10 kV.

In addition, the surface topographies (*n* = 7 per group) were measured by a confocal white light interferometer (UP-Lambda, RTEC instrument, San Jose, CA, USA). An area of 1.6 mm × 1.2 mm on each sample surface was optically scanned. Afterward, the obtained data were transferred to surface analysis software (MountainsMap Universal 9, Digital Surf, Besancon, France). A Gaussian Filter (ISO 16610-61) with a cut-off value of 0.32 mm was applied to differentiate roughness and waviness. Roughness parameters, including arithmetic mean height (Sa) and dales void volume (Vvv), were calculated based on ISO 25178-2:2012. Finally, the surfaces were 3D reconstructed for visualization.

### 2.4. Shear Bond Strength Test

#### 2.4.1. Fabrication of Bonding Mold

**Figure 1:**
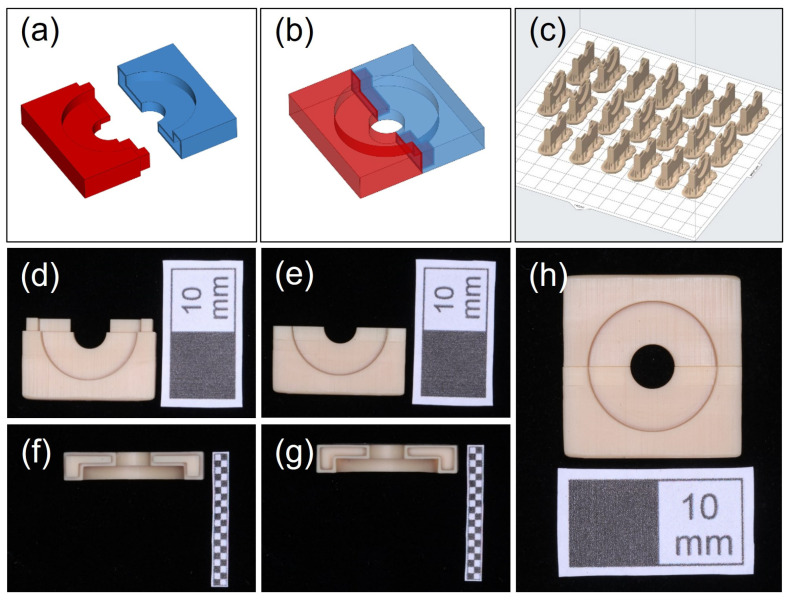
The fabrication of the bonding mold (**a**,**b**). The bonding mold was designed as two parts that can be accurately assembled through a lap joint; (**c**) the direction and layout for 3D printing; (**d**–**h**) the 3D-printed parts could be accurately assembled by lap joint (white scale: 10 mm).

A mold used for bonding the PEEK discs and temporary crown materials was designed by OpenSCAD and Materialise Magics (25.0, Materialise, Leuven, Belgium). The mold consists of two parts (Figure 1a), which can be accurately assembled through a lap joint (Figure 1b). The larger cylindrical space used for placing the PEEK disk was 7.3 mm in radius and 2 mm in height, and the smaller cylindrical space used for adding temporary crown material was 2.5 mm in radius and 2 mm in height. After CAD design, the two parts of the bonding mold were 3D printed by a stereolithography (SLA) printer (Form 3B, Formlabs, Somerville, MA, USA) with a 25 μm-thick layer using model resin (V2, Formlabs). To ensure printing accuracy, the lap joint was placed upward for 3D printing (Figure 1c). After printing, the parts were rinsed with isopropyl alcohol (IPA, Chron chemicals, Chengdu, China) for 10 min in a postcleaning device (Form Wash, Formlabs) and then light cured with 405 nm blue light at 60 °C for 60 min using a postcuring device (Form Cure, Formlabs). Next, the support structures were removed by the finishing kit (Formlabs), and the residues of the support structures were trimmed by a sharp scalpel (Figure 1d–h).

#### 2.4.2. Preparation of the Bonding Specimen

**Figure 2:**
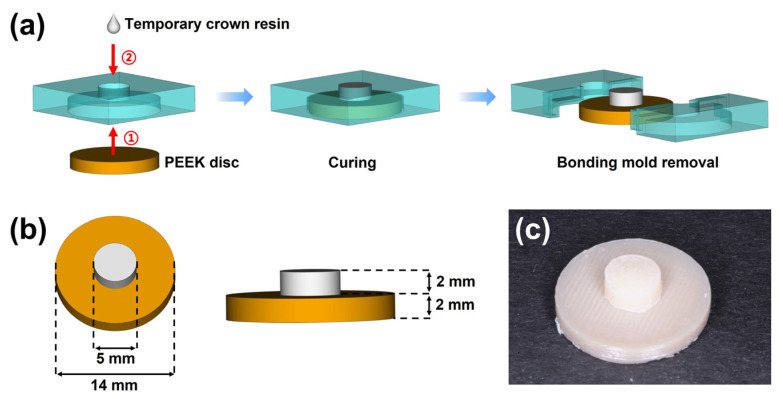
The preparation of the bonding specimen; (**a**) the workflow for fabricating the bonding specimens; (**b**) the dimensions of the bonding specimen after mold removal; (**c**) the fabricated bonding specimen.

Prior to bonding, a thin layer of liquid separating agent (Xin Shi Ji, Shanghai, China) was smeared uniformly on the lateral surface of the smaller cylindrical channel of the bonding mold. Afterward, the PEEK discs were placed in the larger cylindrical space, and three temporary crown resins (*n* = 6 per surface treatment group) were applied for bonding (Figure 2a). All temporary crown materials were cured according to manufacturer’s instructions at 20–22 °C room temperature and 50% relative atmospheric humidity. Table 1 gives detailed information on the temporary crown resins used in the present study. For short, Artificial teeth resin, 3M™ Filtek™ Supreme Flowable Restorative, and Cool Temp NATURAL were denoted as ATR, FR, and CTN, respectively. After curing, the bonding mold was carefully removed (Figure 2b). The fabricated bonding specimens (Figure 2c) were incubated at 37 °C for 24 h in distilled water to simulate the intraoral aqueous environment.

#### 2.4.3. Shear Bond Strength Measurement

**Figure 3:**
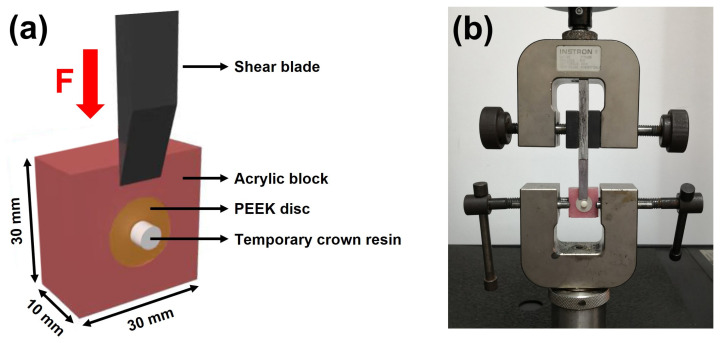
Experimental setup of shear bond strength testing. (**a**) Schematic illustration of the test block; (**b**) shear bond strength test using a universal testing machine.

Before testing, each bonding specimen was fixed in a 30 mm × 30 mm × 10 mm cuboid block made of acrylic resin (Figure 3a). The test block was then immobilized on a universal testing machine (5565, INSTRON, Norwood, MA, USA). A shear load was applied through a blade onto the temporary crown material at a crosshead speed of 1.0 mm/min until debonding (Figure 3b). During measuring, the force and distance were dynamically recorded for plotting the force–distance curves. The shear bond strength was calculated by Equation (1), where *S*, *F*, and *A* represent the shear bond strength (MPa), maximum fracture load (N), and bonding area (mm^2^), respectively.

(1) S=F/A

After measuring, the specimen surfaces were analyzed by a stereoscopic microscope. For further investigation, the debonded samples of the CO_2_ laser group were observed by SEM. Failure modes were determined as prefailure: debonding occurred before the shear bond strength test; adhesive failure: less than 33% of the temporary crown material remained at the bonding interface; mixed failure: more than 33% but less than 66% of the temporary crown material remained at the bonding interface, and cohesive failure: more than 66% of the temporary crown material remained at the bonding interface [21].

### 2.5. Statistical Analysis

All experimental variables were checked for normal distribution by Shapiro–Wilk testing. The roughness data were analyzed by one-way analysis of variance (ANOVA), and Tukey’s test was conducted for the subsequent multiple comparisons. A two-way ANOVA was performed to evaluate the effect of surface treatment and temporary crown material on shear bond strength. In the following main effect analysis, Tukey’s test was used for the posthoc comparisons. GraphPad Prism (9.4.0, GraphPad Software, San Diego, CA, USA) was utilized for all statistical analyses with an α set to 0.05.

## 3. Results

### 3.1. Surface Morphology

**Figure 4:**
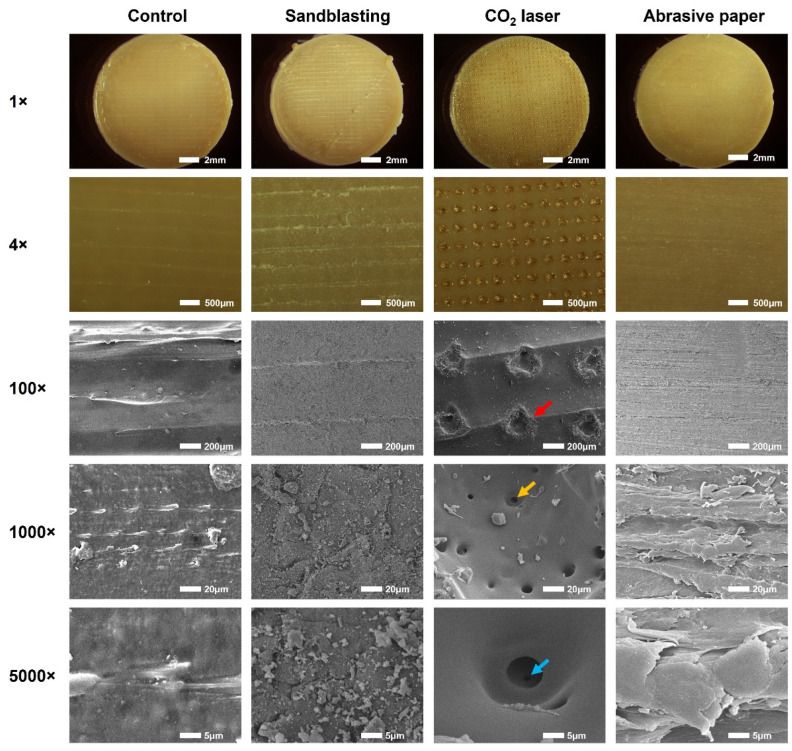
Representative surface morphologies of the 3D-printed PEEK specimens treated with different methods. Images were captured by a stereoscopic microscope (1× and 4×) and a scanning electron microscope (100×, 1000×, and 5000×). The red, yellow, and blue arrows represent the primary, secondary, and tertiary pores created by CO_2_ laser ablation, respectively.

The surface morphologies of the 3D-printed PEEK specimens treated with different methods are shown in Figure 4. The sample surfaces in the control group showed clear textures which were generated from the deposition road of PEEK during FFF printing. In contrast, due to the subtractive surface treatment, these textures became blurred in the sandblasting group and disappeared in the abrasive paper group. CO_2_ lasering did not affect the surface texture but created orderly arranged pores with a diameter of about 200 μm on the PEEK surface (Figure 4, red arrow).

More information was obtained from the highly magnified SEM images (1000× and 5000×). The sample surfaces in the sandblasting group showed collisional traces of Al_2_O_3_ particles, and those in the abrasive paper group exhibited abrasive features generated from silicon carbide grinding. Interestingly, secondary pores with a diameter of approximately 8 μm were found at the bottom and sidewall of the primary pores created by the CO_2_ laser (Figure 4, yellow arrow). In some of the secondary pores, we even detected smaller tertiary pores (Figure 4, blue arrow).

### 3.2. Roughness Measurement

**Figure 5:**
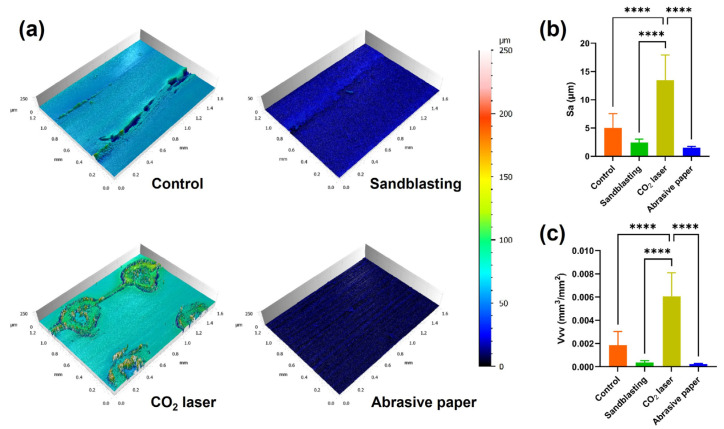
Surface topographies of the 3D-printed PEEK surfaces with various surface treatments; (**a**) the 3D reconstructed surfaces; (**b**) mean (standard deviation) arithmetic mean height (Sa) of each group; (**c**) mean (standard deviation) dales void volume (Vvv) of each group (*n* = 7). Asterisks indicate statistically significant differences (significance level **** *p* < 0.0001).

Figure 5a depicts the reconstructed 3D view of the PEEK surfaces with different surface treatments. The untreated surface (control group) exhibited clear surface textures with a vertical drop of 60 μm. After sandblasting and abrasive paper grinding, the surface textures became blurred or disappeared, which further confirmed the finding in the SEM analysis. The CO_2_ laser created an array of pores on the 3Diprinted PEEK surface. The edge of the pores has risen, which increased the vertical drop to about 80 μm. As shown in Figure 5b,c, the roughness parameter values of Sa and Vvv in the CO_2_ laser group were significantly higher than those of the sandblasting, abrasive paper, and control groups (*p* < 0.0001).

### 3.3. Shear Bond Strength

**Figure 6:**
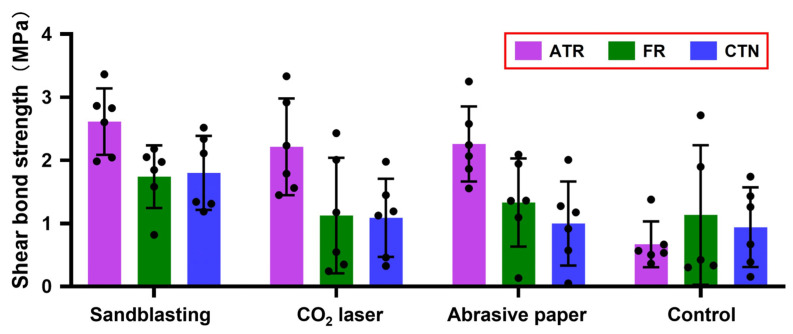
Mean (standard deviation) shear bond strength of each group (*n* = 6). Black dots represent shear bond strength data for each measurement. ATR, FR, and CTN represent Artificial teeth resin, 3M™ Filtek™ Supreme Flowable Restorative, and Cool Temp NATURAL, respectively.

The means and standard deviations of the recorded shear bond strengths are displayed in Figure 6. The results of the two-way ANOVA indicated both surface treatment (F (3, 59) = 8.132, *p* < 0.0001) and temporary crown material (F (2, 59) = 7.885, *p* = 0.0009) had statistically significant effects on shear bond strength, and no obvious interaction (F (6, 59) = 2.166, *p* = 0.0591) could be detected. In the main effect analyses, multiple posthoc comparisons revealed that the shear bond strengths of the sandblasting group and abrasive paper group were significantly higher than that of the control group (*p* < 0.05), and the shear bond strength generated by ATR was significantly higher than those produced by FR and CTN (*p* < 0.01).

**Figure 7:**
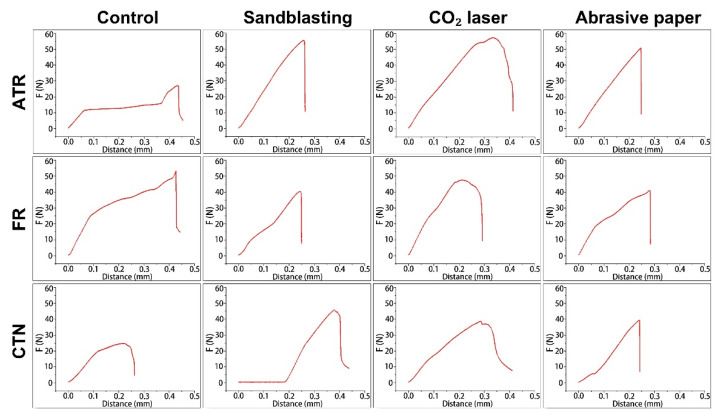
Representative force-distance curves of each group in shear bond strength testing. ATR, FR, and CTN represent Artificial teeth resin, 3M™ Filtek™ Supreme Flowable Restorative, and Cool Temp NATURAL, respectively.

Figure 7 shows the representative force-distance curves of each group. Generally, the curves started to ascend rapidly once the shear blade contacted the bonding specimen. After the shear blade moved about 0.2–0.3 mm, the curves reached the peak. From this point, the detachment between the temporary crown materials and PEEK discs occurred, and the curves dropped quickly. When compared with the sharp peaks in the control, sandblasting, and abrasive paper groups, the peaks in the CO_2_ laser group were round and blunt.

### 3.4. Failure Mode Analysis

**Figure 8:**
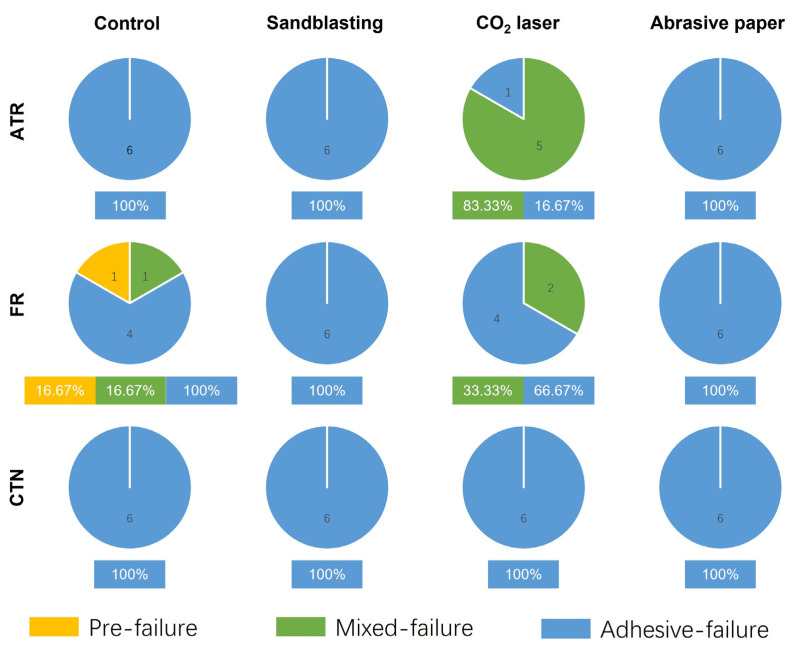
The failure mode for each experimental group after shear bond testing (*n* = 6). ATR, FR, and CTN represent Artificial teeth resin, 3M™ Filtek™ Supreme Flowable Restorative, and Cool Temp NATURAL, respectively.

Figure 8 illustrates the composition of the failure mode of each group after the shear bond strength test. Adhesive failures were mostly observed except for the ATR-CO_2_ laser group, whose failure mode was dominated by mixed failure (83.33%). In addition, mixed failure could also be found in the FR-CO_2_ laser group (33.3%) and the FR-Control group (16.67%). One bonding specimen in the FR-control group exhibited prefailure before shear bond testing. The FR material debonded from the untreated 3D-printed PEEK surface during incubation in distilled water.

**Figure 9:**
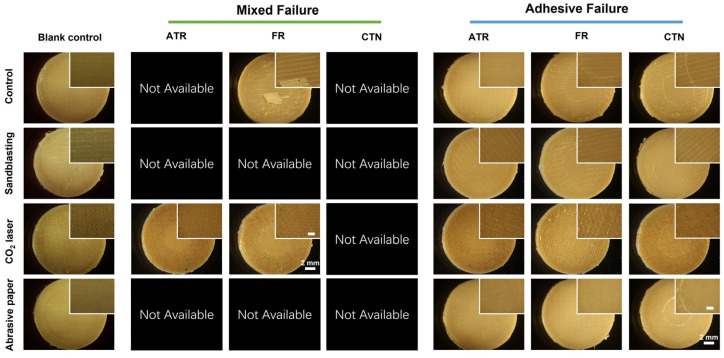
Representative sample surfaces after shear bond strength testing. Images were collected by a stereoscopic microscope at 1× and 4× magnifications. The scale bar in the zoomed figure is 1 mm. ATR, FR, and CTN represent Artificial teeth resin, 3M™ Filtek™ Supreme Flowable Restorative, and Cool Temp NATURAL, respectively.

Figure 9 shows the sample surfaces after shear bond testing. In terms of adhesive failure, the rapture site was located at the interface between PEEK and the temporary crown material. The residual resin could hardly be observed on the PEEK surfaces.

With regard to mixed failure, a large piece of FR material was found on the untreated PEEK surface. In contrast, residual resin pieces could not be found on the sample surfaces treated by CO_2_ laser. The ATR and FR material remained in the pores created by CO_2_ laser ablation, which could be further proved by SEM observation.

**Figure 10:**
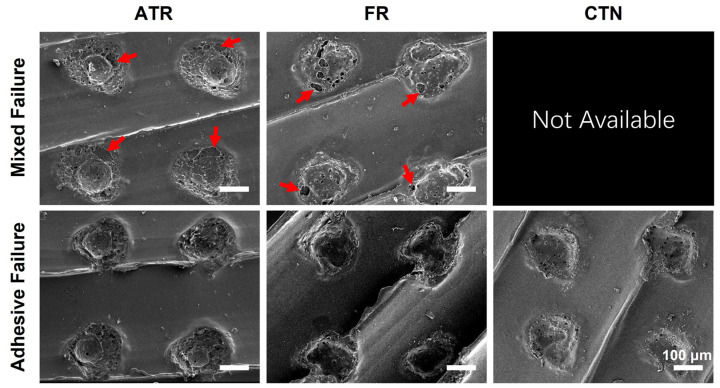
Representative SEM images of the samples treated by CO_2_ laser after shear bond strength testing (130×). Red arrows indicate the voids in the residual temporary crown resins. ATR, FR, and CTN represent Artificial teeth resin, 3M™ Filtek™ Supreme Flowable Restorative, and Cool Temp NATURAL, respectively.

The results of the SEM exhibit that the residual ATR and FR material remained in the pores created by the CO_2_ laser (Figure 10, mixed failure). Interestingly, the material residuals were not solid. Voids were detected at the periphery and interior of the material residuals (Figure 10, red arrow).

## 4. Discussion

Since its development in 1978 by British chemists, PEEK has attracted increasing attention in the biomedical field due to its excellent mechanical properties [22]. Being chemically inert, PEEK is also deemed a cost-effective alternative to metallic materials used in dentistry [7,23]. The recent development of 3D printing technologies facilitates the fabrication of customized dental prostheses [24]. However, for temporary abutment application, less is known for the adhesive property of 3D-printed PEEK material, especially its bonding with temporary crown materials. According to the shear bond strength test results, both surface treatment and temporary crown material had statistically significant effects on shear bond strength. Therefore, the null hypotheses were rejected.

The results of the SEM (Figure 4) and 3D reconstruction (Figure 5a) indicated that the surfaces of the 3D-printed PEEK samples were not smooth. Our previous study reported that roughness could be generated by the unfilled area between the layers deposited by FFF printers [11]. However, in this study, the roughness was found within a layer on the top surface, so the mechanism behind roughness generation is not the same as before. FFF 3D printers work by extruding molten materials out of a heated nozzle [25]. During the printing of the last layer (top surface), the nozzle repeatedly moves along paths in one direction to deposit materials until the entire surface is covered. In this process, unfilled voids can be found among the paths, generating the surface texture and roughness observed in this study [26]. In clinical practice, sandblasting and abrasive paper are commonly used to roughen surfaces before bonding. However, for a sufficiently rough surface, subtractive surface treatments may only smooth it [11]. This could be verified by the blurred or disappeared surface textures in the sandblasting and abrasive paper group (Figure 4 and Figure 5a). The CO_2_ laser generator could emit high-energy beams of photons at a wavelength of 10600 nm from the medium of carbon dioxide gas [27]. Focused CO_2_ laser beams produce ultra-high energy density at a convergence point [28]. In the present study, the CO_2_ laser array was composed of numerous focused laser beams. These laser beams ablated the PEEK material, creating an array of pores on the surface (Figure 4 and Figure 5a). The second and tertiary pores might be generated by the instant vaporization effect. However, this speculation needs further research for clarification.

This study selected two areal roughness parameters that might be related to bonding for investigation. Sa is the most frequently used areal roughness parameter that characterizes surface height, and Vvv is a 3D functional parameter used for evaluating the void volume at the valley zone. The results of the roughness measurements indicated that Sa and Vvv had similar changes after surface treatments (Figure 5b,c). Sandblasting and abrasive paper grinding slightly reduced the mean value of Sa and Vvv but did not produce statistically significant differences. This is consistent with the results of the SEM analysis. In contrast, CO_2_ laser ablation substantially enhanced surface roughness, which could be attributed to the pores generated on the surface.

Currently, there are no acknowledged clinical success criteria for the bond strength between temporary abutment and crown because implant prostheses may undergo complex stresses that are generated from mastication. Nevertheless, the bond strength should be optimized to avoid clinical failure. The results of the shear bond strength tests in this study indicated that ATR had a statistically higher shear bond strength with 3D-printed PEEK surfaces when compared to FR and CTN. This might be explained by the fact that the main components of FR and CTN are multifunctional acrylates (Table 1), which may induce higher volume shrinkage during polymerization [29]. As mentioned above, the PEEK specimens treated with the CO_2_ laser seemed to possess surfaces that were better for bonding. Interestingly, the shear bond strength test in this study produced the opposite results, indicating that surface roughness may not be the only decisive factor in shear bond strength. The possible explanation could be related to the decreased wettability. The correlation between roughness and wettability conforms the Wenzel Equation (2) defined in 1936 [30]:

(2) $$\cos\theta_{1}=r\cos\theta_{2}$$

where $\theta_{1}$, $\theta_{2}$, and *r* represent the apparent contact angle, Young’s contact angle, and roughness ratio, respectively. For a hydrophobic PEEK surface, an increase in surface roughness can make the surface even more hydrophobic. Previous studies found that laser treatments could significantly increase the water contact angle on PEEK surfaces to about 110°, turning PEEK surfaces into a highly hydrophobic state [31,32,33,34]. In addition to the increased surface roughness, Akkan et al. attributed the increased hydrophobicity to the modification in surface topology [32]. Similarly, Riveiro et al. indicated that the microstructures created by laser ablation could significantly decrease the wettability of PEEK surfaces [34]. It should be noted that the liquid resins used in this study are different from the ultrapure water used for the standard wettability tests in previous studies. Due to the curing from monomer to polymer, the flowability of liquid resins may decrease rapidly during wetting. This phenomenon could further inhibit liquid resins from spreading out on the laser-treated PEEK surfaces, creating unfilled voids at the bonding interface. The above speculations are demonstrated by the results of the failure mode analysis (Figure 10). Void areas were mainly detected at the resin–PEEK interface. In order to further improve the bonding between the 3D-printed PEEK and the temporary crown materials, attempts could be focused on simultaneously improving surface roughness and wettability. Effective ways to enhance surface energy, such as atmospheric plasma [35], deserve further research for a full re-evaluation.

In previous studies, the fabrication of bonding specimens was considered to be time-consuming. The conventional method utilized a specially made metallic mold for sample preparation [8,36]. However, not all labs have access to such equipment, and researchers may fail to fabricate standardized specimens due to poor laboratory conditions. In addition, in order to use the conventional metallic mold, base materials (the material for investigation) must be accurately tailored into a specific dimension, which further increases the difficulties in experiments. More importantly, after bonding, the removal of the metallic mold was along the longitudinal axis of the bonded cylinder material. In this process, the bonded material might be debonded from the base material, and if not, microdetachment may also occur at the bonding interface, affecting shear bond strength. With the help of CAD and AM, this study fabricated customized split molds for sample preparation. This method is not limited by the conventional metallic mold or the sample size. After bonding, the removal of the split mold occurs from the lateral side of the bonded cylinder material, which minimizes the influence of mold removal on the shear bond strength measurement.

At present, different adhesives are being developed to improve the bonding performance of PEEK [37]. The authors assume that the additional use of adhesives may produce higher bond strengths. However, for experimental standardization, we did not include these adhesive systems, which deserves further research for clarification. Another limitation of the present study is that the effect of polymer aging in an oral-aqueous environment on shear bond strength was not considered. For future studies, conducting thermal cycling to simulate the intraoral aging process [38] might be more effective in predicting the long-term adhesive property of additively manufactured PEEK materials.

## 5. Conclusions

This study investigated the effects of surface treatment and temporary crown material on the shear bond strength of a 3D-printed PEEK material. Our results indicated that surface roughness might not be the only factor in determining the adhesive property of additively manufactured PEEK. For clinical application, the ATR material and subtractive surface treatments (sandblasting and abrasive paper grinding) are recommended for 3D-printed PEEK abutment to obtain a higher shear bond strength.

## Figures and Tables

**Table 1:** The temporary crown resins used for shear bond strength testing.

Product Name	Manufacturer, City, Country	Lot No.	Main Component
Artificial teeth resin	Xin Shi Ji, Shanghai, China	20211118	Methyl methacrylate
3M™ Filtek™ Supreme Flowable Restorative	3M, St. Paul, MN, USA	NF20341	Dimethacrylates
Cool Temp NATURAL	Coltène/Whaledent AG, Altstätten, Switzerland	L25817	Trimethylolpropane trimethacrylate, dimethacrylates

## Data Availability

Not applicable.
